# Long-Term Adherence to IFN Beta-1a Treatment when Using RebiSmart® Device in Patients with Relapsing-Remitting Multiple Sclerosis

**DOI:** 10.1371/journal.pone.0160313

**Published:** 2016-08-15

**Authors:** O. Fernández, R. Arroyo, S. Martínez-Yélamos, M. Marco, J. A. García Merino, D. Muñoz, E. Merino, A. Roque, RELOAD Study Group

**Affiliations:** 1 Hospitales Universitarios Regional de Málaga y Virgen de la Victoria, Universidad de Málaga, IBIMA; 2 Hospital U. Clínico San Carlos, Madrid; 3 Hospital Universitario de Bellvitge, Barcelona; 4 Corporació Sanitaria Parc Taulí, Barcelona; 5 Hospital Universitario Puerta de Hierro, Madrid; 6 Hospital Xeral Cies, Vigo; 7 Departamento Médico, Merck S.L; Charite Universitatsmedizin Berlin, GERMANY

## Abstract

The effectiveness of disease-modifying drugs in the treatment of multiple sclerosis is associated with adherence. RebiSmart^®^ electronic device provides useful information about adherence to the treatment with subcutaneous (sc) interferon (IFN) β-1a (Rebif^®^). The aim of the study was to determine long-term adherence to this treatment in patients with relapsing-remitting multiple sclerosis (RRMS). This retrospective multicentre observational study analysed 258 patients with RRMS who were receiving sc IFN β-1a (Rebif^®^) treatment by using RebiSmart^®^ until replacement (36 months maximum lifetime) or treatment discontinuation. Adherence was calculated with data (injection dosage, time, and date) automatically recorded by RebiSmart^®^. Patients in the study had a mean age of 41 years with a female proportion of 68%. Mean EDSS score at start of treatment was 1.8 (95% CI, 1.6–1.9). Overall adherence was 92.6% (95% CI, 90.6–94.5%). A total of 30.2% of patients achieved an adherence rate of 100%, 80.6% at least 90%, and only 13.2% of patients showed a suboptimal adherence (<80%). A total of 59.9% of subjects were relapse-free after treatment initiation. Among 106 subjects (41.1%) who experienced, on average, 1.4 relapses, the majority were mild (40.6%) or moderate (47.2%). Having experienced relapses from the beginning of the treatment was the only variable significantly related to achieving an adherence of at least 80% (OR = 3.06, 1.28–7.31). Results of this study indicate that sc IFN β-1a administration facilitated by RebiSmart^®^ could lead to high rates of adherence to a prescribed dose regimen over 36 months.

## Introduction

Multiple sclerosis (MS) is an autoimmune and chronic disease characterized by inflammation, demyelination and axonal degeneration occurring in the central nervous system [1,2]. Most patients are between 25–35 years of age when diagnosed, and they are mainly women. The prevalence in Spain ranges around 70–80 to 125 cases per 100,000 inhabitants, depending on the geographical area, and the used methodology [3,4]. Approximately 70–80% of patients develops the relapsing-remitting form (RRMS), defined by recurrent episodes of neurological dysfunction, and followed by partial or full recovery [5]. Although to date there is no cure for MS, first-line treatment with disease-modifying drugs (DMDs), such as interferon (IFN) β-1a, IFN β-1b, and glatiramer acetate, have demonstrated to reduce the incidence and severity of relapses and disability progression [6–9]. Apart from this partial effect, the efficacy of treatments is being compromised by the lack of persistence and adherence to the prescribed duration, interval and dosing [10,11]. Patients with poor adherence, or who discontinued the treatment, have reported higher incidence of relapses and experienced worse quality of life than adherent patients [12–15]. Indeed, adherence to injectable DMD therapies is generally suboptimal [11].

The Global Adherence Project (GAP), a multicentre, multinational phase IV study that involved 2,566 patients with RRMS, revealed a non-adherence rate of 25% to prescribed regimens (intramuscular IFN β-1a, subcutaneous, sc, β-1a 22 and 44μg, IFN β-1b, and glatiramer acetate) [15]. In another study performed in Spain [16], the proportion of adherent patients to sc IFN β-1b, assessed by the Morisky-Green test, was 68.3%, being indicative of poor adherence. Primary reasons described for non-adherence include adverse reactions (AEs), ‘flu-like symptoms’, injection anxiety, perceived lack of efficacy, or forgetfulness [15–19]. Among the strategies aimed at improving the adherence to sc IFN β-1a treatments is the use of autoinjection devices, such as RebiSmart^®^ (Merck Serono SA—Geneva, Switzerland), which also increase significantly the satisfaction of patients [20,21]. In BRIDGE (RebiSmart^®^ to self-inject Rebif^®^ serum-free formulation in a multidose cartridge), a 12-week study involving 119 patients with RRMS, the adherence rate was 88.2% [22]. Despite these encouraging short-term results, and with the unique exception of a recent published study [23], to date there is no study evaluating the long-term adherence to sc IFN β-1a treatments in clinical practice. Furthermore, no study has been conducted in Spain with this drug. Therefore, the aim of the present study was to determine long-term adherence to sc IFN β-1a treatment administered with the RebiSmart^®^ device in patients with RRMS.

## Materials and Methods

**Fig 1:**
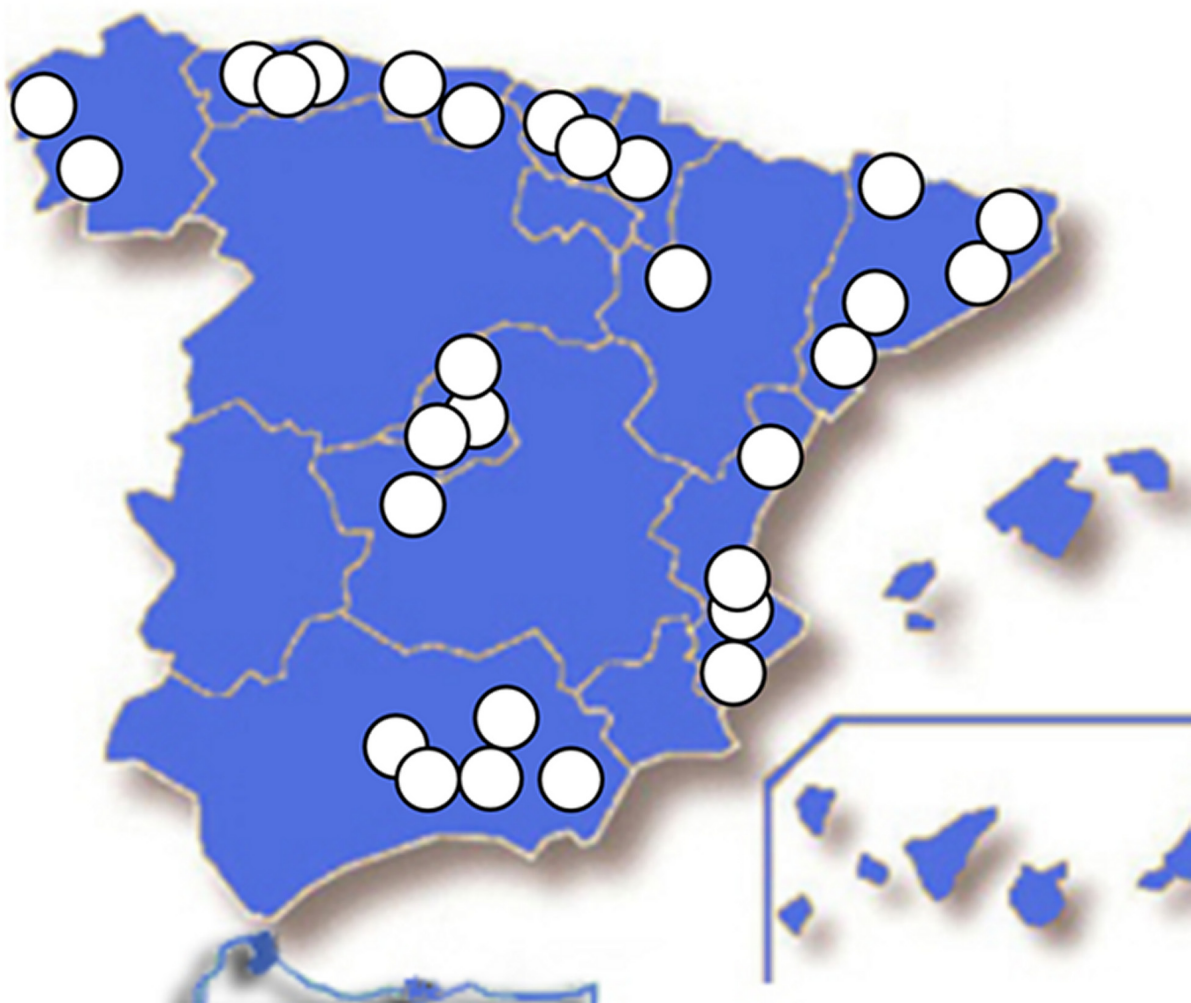
Geographical location of the hospitals participating in the RELOAD study. This map of Spain shows the localization of the 29 centres (green circles) that participated in the present study. Picture modified from: https://es.wikipedia.org/wiki/Archivo:Espa%C3%B1aLoc.svg

This retrospective, multicentre, observational nationwide study analysed 258 patients with RRMS who were receiving sc IFN β-1a treatment (Rebif^®^) by using RebiSmart^®^ device. This study was carried out in 29 hospitals from Spain (Fig 1).

Criteria for study inclusion were as follows: aged 18 or over; diagnosed with RRMS; receiving sc treatment with IFN β-1a by means of RebiSmart^®^; using RebiSmart^®^ device until replacement (36 months maximum lifetime) or treatment discontinuation, by any cause; electronic download of adherence data stored in the RebiSmart^®^ device (using the Mitra^®^ software version 1.5) from start of treatment to replacement/return of the RebiSmart^®^ device; and signed informed consent. Exclusion criteria were: having been diagnosed with clinically isolated syndrome, or having primary or secondary progressive MS with no relapses. Patients were invited to participate in the study at the time of a scheduled visit for device replacement. The recruitment window for collecting RebiSmart^®^ devices was 6 months, from June 2013 to December 2013. Moreover, patients who discontinued the treatment and returned the device between October 2009 and August 2013 were also invited to participate in the study. Procedures were performed in accordance with guidelines established by the Ethics Committee of each participating center, and the Declaration of Helsinki.

### Study endpoints

The primary assessment was adherence to the treatment over the retrospective period of observation, i.e. from the start of sc IFN β-1a treatment to the time of device replacement or treatment discontinuation. Overall adherence was calculated as follows:

$$100\;\text{X}\;\frac{\text{total number of sc IFN}\;\beta \text{-}1\text{a administrations}}{\text{total number of days}}\;\text{X}\;\frac{7\;\text{days}}{3\;\text{administrations}}$$

Adherence was quantified by using the data (dosage, time, and date) automatically recorded by RebiSmart^®^. The primary endpoint was overall adherence (as continuous outcome) supported by the secondary endpoint, percentage of subjects with at least ≥60%, ≥70%, ≥80%, ≥90%, and 100% adherence.

Data from adherence were analysed by using the overall period of study and by quarters. Secondary endpoints included clinical and demographic characteristics of subjects treated with sc IFN β-1a by using RebiSmart^®^, the evaluation of disease relapses during the study period, treatment discontinuation including reasons, and subsequent treatments after discontinuation. Disease relapse was defined as the reappearance of neurological symptoms during at least 24 hours after a period of time with stable neurological status or after at least 30 days of improvement. Relapses were classified according to the severity (mild, moderate, or sever) of the episodes. The Expanded Disability Status Scale (EDSS) score was estimated at the time of starting sc IFN β-1a treatment and when included in the study at the time of device replacement or treatment discontinuation.

### Statistical Analysis

Qualitative variables were expressed as absolute and relative frequencies, and quantitative variables as the mean with the standard deviation (SD) or the 95% confidence interval (95% CI). Confidence intervals for rates of adherence were calculated by using Wilson score and Clopper-Pearson (Exact) method. A repeated measures analysis of variance (ANOVA) was performed to evaluate differences in overall adherence over time. Statistical significance was established when p≤0.05. A logistic regression model was estimated to identify demographic and clinical characteristics of patients (including age, sex, geographic location, using a different IFN β-1a as first DMD treatment, number and severity of relapses, and duration of the treatment) related to achieving an adherence ≥80%. All statistical analyses were performed by using SAS software 9.2.

## Results

A total of 276 patients were recruited in the study, but only 258 fulfilled all inclusion criteria and were thus included in the analysis. The study population consisted of mainly women (67.8% of patients), Caucasian patients (98.8%), with a mean age of 40.7 years and mean body mass index of 23.9 Kg/m^2^. Demographic and clinical characteristics of subjects are shown in Table 1.

**Table 1:** Demographic and clinical characteristics of patients.

	Total(N = 258)
**Sex**, female, n (%)	175 (67.8)
**Race**, Caucasian, n (%)	255 (98.8)
**Age**, mean years (SD)	40.7 (9.5)
**BMI**, mean Kg/m^2^ (SD)	23.9 (3.8)
**Employment status**, n (%)	
Employed	134 (51.9)
Unemployed	33 (12.8)
Retired	24 (9.3)
Unpaid housework	14 (5.4)
Student	7 (2.7)
Not available	46 (17.8)
**Educational level**, n (%)	
Primary	41 (15.9)
High school	90 (34.9)
University	75 (29.1)
No studies	1 (0.4)
Not available	51 (19.8)
**Requiring a caregiver**, n (%)	10 (3.9)
**Time since diagnosis of RRMS**, mean years (SD)	8.9 (6.0)
**Time since onset of symptoms**, mean years (SD)	11.3 (6.9)
**Patients using sc IFN β-1a as first DMD treatment**, n (%)	211 (81.8)
Time since the start of the treatment, mean years (SD)	3.1 (0.8)
**Patients with relapses since the beginning of the treatment**, n (%)	106 (41.1)
Number of relapses, mean (95% CI)	1.4 (1.2–1.6)
Maximum severity of relapse: Mild, n (%)	43 (40.6)
Moderate, n (%)	50 (47.2)
Severe, n (%)	6 (5.7)
Not available, n (%)	7 (6.6)
**EDSS score**, mean (SD)	
At start of sc IFN β-1a treatment (baseline)	1.8 (1.2)
At inclusion in the study (last time point)	2.0 (1.6)

SD, standard deviation; BMI, body mass index; RRMS, relapsing-remitting multiple sclerosis; sc, subcutaneous; DMD, disease modifying drug; EDSS, Expanded Disability Status Scale

Half of patients (51.9%) were employed at the time of inclusion. A total of 34.9% completed the high school and 29.1% the university. Only 3.9% of patients required a caregiver for assistance. The mean time since diagnosis of RRMS and from onset of symptoms was 8.9 years and 11.3 years respectively. Most of subjects (81.8%) had been using sc IFN β-1a as first DMD treatment. Patients have been receiving Rebif^®^ with RebiSmart^®^ device for 3.1 years. Since the beginning of the IFN β-1a treatment, 106 patients (41.1%) had experienced on average 1.4 relapses (SD: 1.0), with maximum severity being mainly moderate (47.2% of subjects with relapses) or mild (40.6%). Mean EDSS score was 1.8 (SD: 1.2) at the time of sc IFN β-1a treatment onset and 2.0 (SD: 1.6) at the study inclusion visit (time of device replacement or treatment discontinuation).

**Fig 2:**
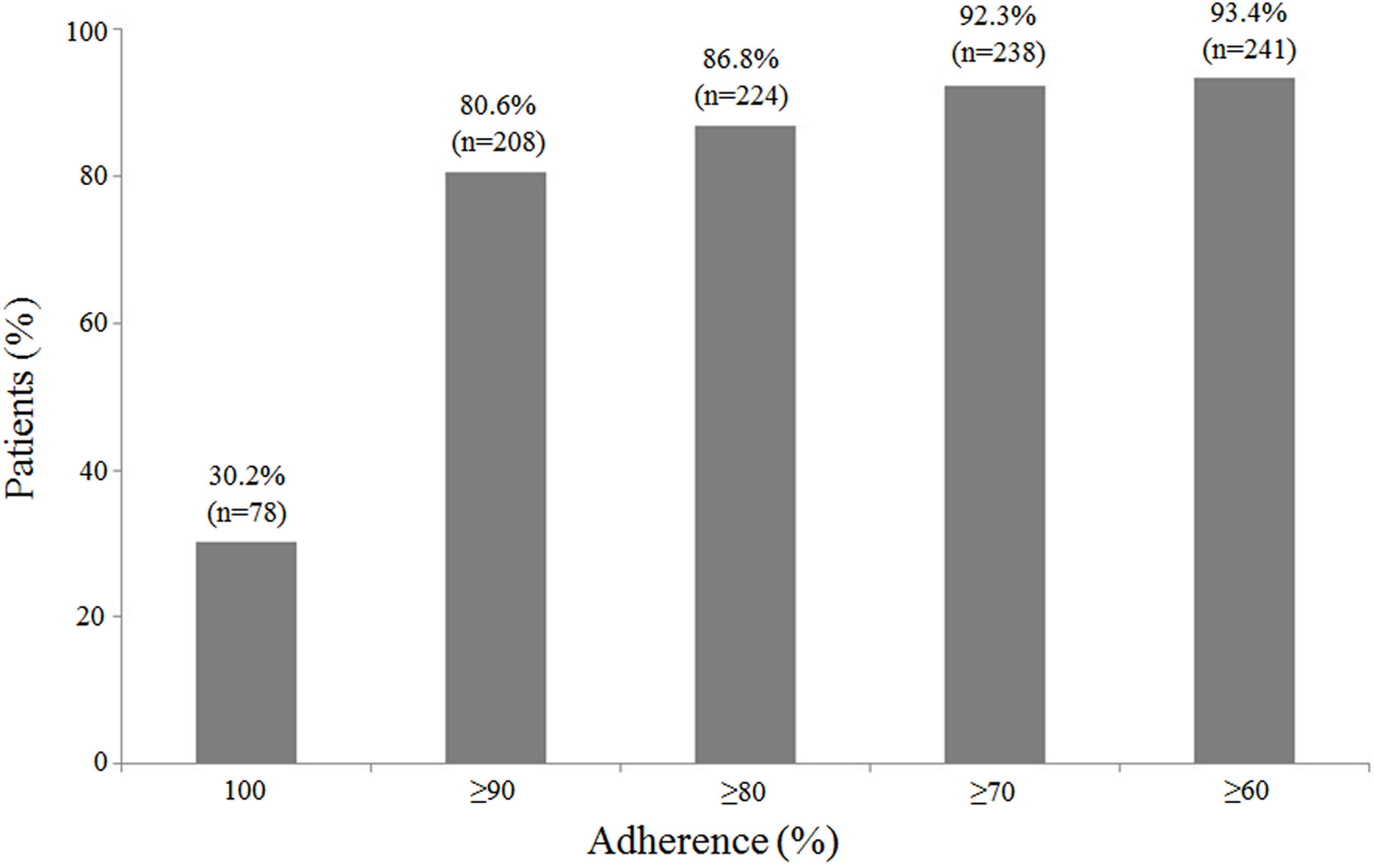
Adherence to the treatment. Percentage of patients achieving (≥60%, ≥70%, ≥80%, ≥90%, or 100% adherence to sc IFN β-1a treatment over 36 months of the study period (N = 258). The number of patients and the respective percentage is depicted above each bar.

Overall adherence during the study period was 92.6% (95% CI: 90.6–94.5%). A total of 78 subjects (30.2%) achieved an adherence rate of 100%, whereas 208 (80.6%) achieved a rate of at least 90% (Fig 2).

**Fig 3:**
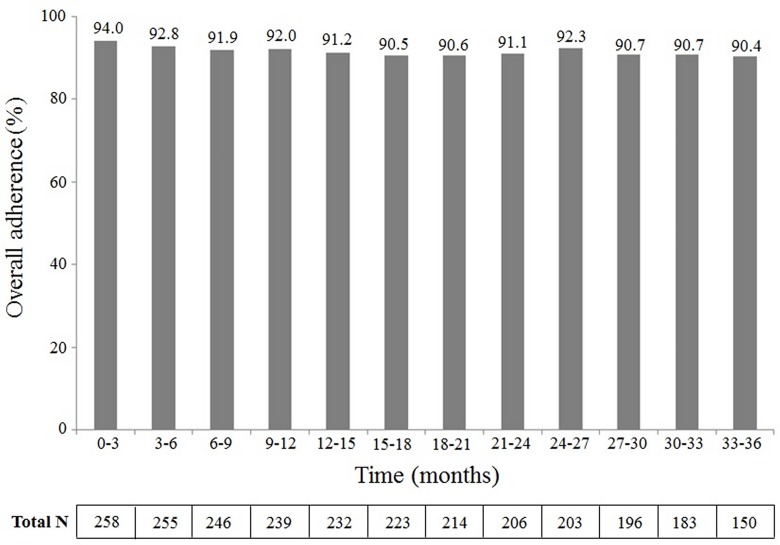
Adherence to the treatment by quarters. Overall adherence to sc IFN β-1a treatment over 36 months of the study period and analysed by quarters. The mean value of the overall adherence in each quarter is depicted above the respective bar. The total number (N) of patients included in each quarter is shown below the graphic.

Only 34 subjects (13.2%) showed a suboptimal adherence (<80%) during the study period. When analysed by quarters (Fig 3), overall adherence at the beginning of the treatment (0–3 months) showed the maximum value (mean: 94.0; 95% CI: 92.0–96.0).

From there, overall adherence decreased slightly until 90.4; 95% CI: 87.4–93.3) at the time of device replacement (n = 150). No significant differences were found between rates of adherence through the study period. The percentage of subjects with suboptimal adherence by 3 months periods was about 10% throughout the study period (range: 8.1–13.2%).

**Fig 4:**
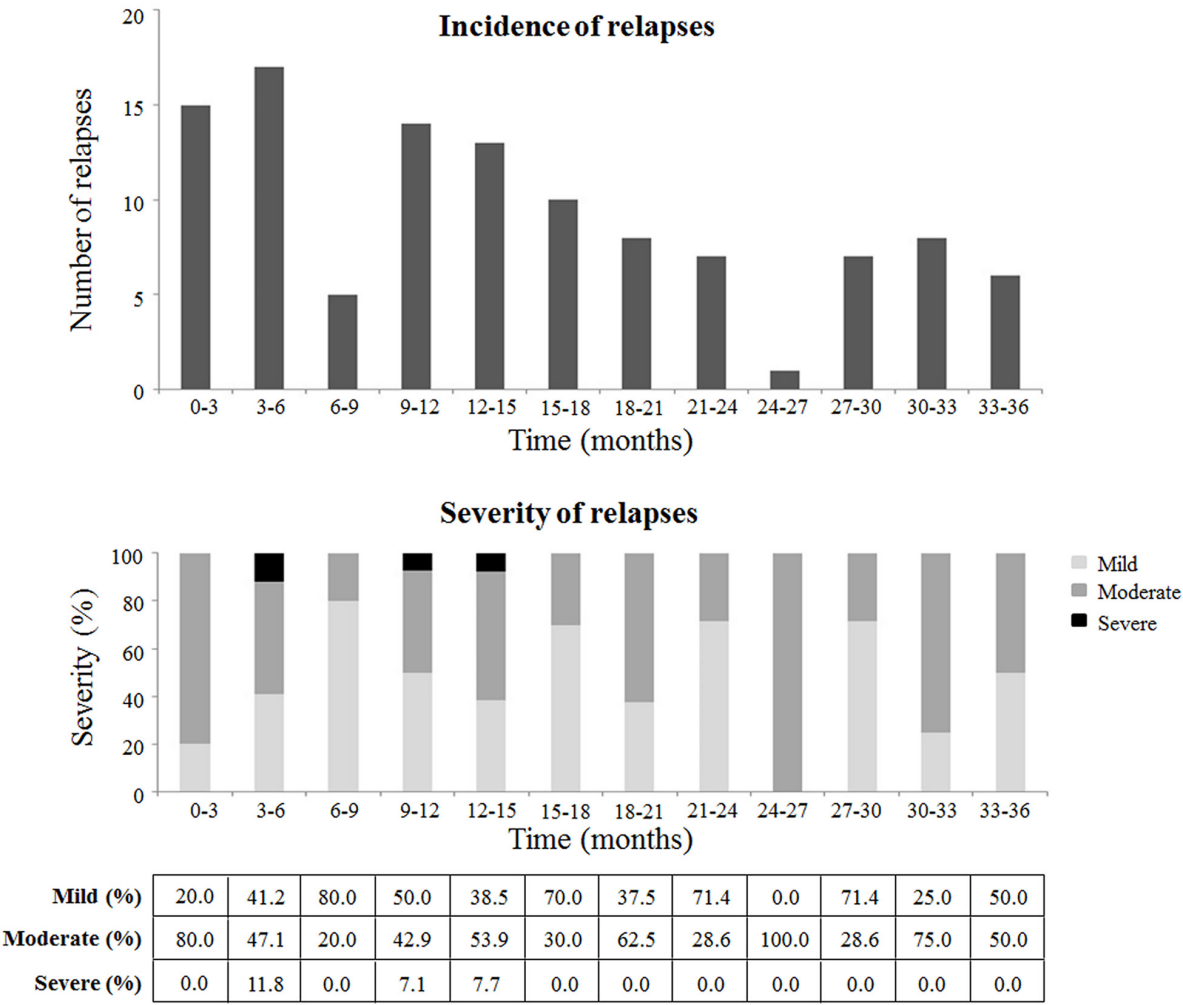
Relapses occurred during the treatment. Incidence (upper graph) and severity (lower graph) of relapses over 36 months of the study period and analysed by quarters. The incidence of relapses is analysed by determining the mean number of relapses in each quarter of time. The severity of relapses in each quarter of time is classified in mild (light grey portion of the bar), moderate (dark grey), or severe (black). The percentage of mild, moderate and severe relapses experienced in each quarter is shown below the graphic.

According to the logistic regression model, having experienced relapses from the beginning of the sc IFN β-1a treatment was the only variable associated with achieving an adherence of at least 80% (OR: 3.06, 95% CI: 1.28–7.31). The incidence of relapses decreased over time, from 15 cases at the beginning of the treatment (5.8%, n = 258 at 0–3 months) to 6 cases at the time of device replacement (4.0%, n = 150 at 33–36 months; Fig 4). Relapses were mainly mild or moderate in severity.

A total of 89 patients (34.5%) discontinued the treatment before device replacement mainly due to the following reasons: occurrence of AEs (12.8% of patients), perceived lack of efficacy (10.8%), voluntary decision (8.1%), and non-adherence to the treatment (6.2%). Discontinuation of the treatment, reasons, and subsequent treatments are shown in Table 2.

**Table 2:** Discontinuation of the treatment, reasons, and subsequent treatments.

	Total
**Total number of patients that discontinued**, n/total N (%)	89/258 (34.5)
0–3 months	5/258 (1.9)
3–6 months	10/253 (4.0)
6–9 months	7/243 (2.9)
9–12 months	10/236 (4.2)
12–15 months	11/226 (4.9)
15–18 months	10/215 (4.7)
18–21 months	8/205 (3.9)
21–24 months	7/197 (3.6)
24–27 months	5/190 (2.6)
27–30 months	6/185 (3.2)
30–33 months	2/179 (1.1)
33–36 months	8/177 (4.5)
**Rate of discontinuation each quarter**, mean (95% CI)	3.4 (2.7–4.1)
**Main reasons for discontinuation**, n/total N (%)	
Occurrence of adverse events	33/258 (12.8)
Perceived lack of efficacy	28/258 (10.8)
Voluntary decision	21/258 (8.1)
Non-adherence	16/258 (6.2)
**Main subsequent treatments after discontinuation**, n /N discontinued (%)	
Glatiramer acetate	20/89 (22.5)
Fingolimod	12/89 (13.5)
Natalizumab	11/89 (12.4)
IFN β-1a intramuscular	8/89 (9.0)

95% CI, 95% confidence interval

The occurrence of AEs was 15 cases during the first year (5.8%, N = 258), 13 during the second (5.6%, N = 233), and 5 during the third (2.5%, N = 203). Discontinuation remained at a constant rate through the study period, with a mean value of 3.4% of subjects (95% CI: 2.7–4.1%) each quarter. Finally, main treatments after discontinuation were: glatiramer acetate (22.5% of the changes, 20/89), fingolimod (13.5%, 12/89), natalizumab (12.4%, 11/89), and IFN β-1a intramuscularly (9.0%, 8/89).

## Discussion

Despite the demonstrated clinical effectiveness of DMDs by reducing the incidence and severity of relapses and disability progression in RRMS [4–7,24], adherence to injectable DMD therapies is generally suboptimal [10], which compromises their potential beneficial effects. Data from the GAP project revealed an overall non-adherence rate of 25% to intramuscular IFN β-1a, sc IFN β-1a, IFN β-1b, and glatiramer acetate first-line DMD treatments [15]. Moreover, adherent patients showed a significant better quality of life, fewer neuropsychological issues, shorter duration of disease, and shorter duration of therapy than non-adherent patients. These results highlighted the importance of adherence on the effectiveness of the DMD treatments and the need of identify the causes that lead to non-adherence. The most common reported reasons for non-adherence included forgetting to administer the injection, followed by tiredness of taking injections, fatigue, ‘flu-like symptoms’, and pain.

Maintaining the adherence to injectable treatments may be a challenge for some patients when managing a chronic disease. Electronic injection devices, such as RebiSmart^®^, have been developed with the purpose to overcome specific problems associated with the injection (needle phobias, anxiety, or pain at the injection site) [21,25,26]. When comparing with manual injections, autoinjectors have demonstrated to improve the injection tolerability and thus the satisfaction of the subject [27–30]. For this reason, the advance of these electronic injection devices has led to the improvement of adherence to sc IFN β-1a treatment, as demonstrated in the 12-week BRIDGE study, with a rate of adherence of 88.2% [22]. Subjects reacted favourably to those features of the device associated with handling, while being easy to use. Similarly, it has been recently published a study of adherence over 24 months in 225 patients with RRMS from the United Kingdom (UK) and Ireland who were receiving sc IFN β-1a treatment by RebiSmart^®^ [23]. In this population, the rate of overall adherence was 95.0%, showing 92.0% of subjects with an adherence ≥80% at 12 months and 91.1% at 24 months. In our study, the overall rate over the course of 36 months (92.6%) was in concordance with previous studies using the same RebiSmart^®^ device, corroborating its beneficial effect on improving the adherence to the treatment. In our study, the only demographic and clinical variable from subjects related with an adherence of at least 80% was having (or not) experienced relapses from the beginning of the sc IFN β-1a treatment. Indeed, suboptimal adherence was about 3 times higher in subjects who had suffered relapses than in those who had not. Furthermore, 58.9% of the subjects did not experience any relapse since the beginning of the treatment, thus corroborating its effectiveness in reducing the incidence of relapses [4–8]. A total of 34.5% of subjects discontinued the treatment before device replacement because of the occurrence of adverse events, perceived lack of efficacy, voluntary decision, or non-adherence. In our study, discontinuation remained at a constant rate through the study period, in contrast to what has been reported in the literature, occurring more likely in the first 6 months of treatment [10]. Those subjects who discontinued the treatment, mainly switched to glatiramer acetate, or to the second-line agents fingolimod or natalizumab. RebiSmart^®^ device also can record the dose history, allowing the capture of accurate and objective information, useful to detect suboptimal adherence in patients and to avoid potential lack of efficacy. This feature becomes especially important for patients with memory or attention impairment. The present study has several strengths. It evaluated the effect of using an electronic device which may inform about the adherence to disease-modifying therapies. This fact allows avoiding healthcare database as a source of information. Therefore, this device is able to monitor adherence objectively in contrast to database studies, using medication possession ratio. The main limitation of the study was its retrospective nature. Furthermore, there is the possibility of a selection bias derived from the fact that patients aware of their non-adherence may tend to not participate voluntarily in the study. However, and unfortunately, this potential bias is intrinsically present in all studies in this field. Another issue, is the fact that although the patients were selected by the time of device replacement or treatment discontinuation, the age range was relatively small, and therefore we must accept the possibility that a hidden age selection bias could have been inadvertently occurred, when prescribing the RebiSmart® device, as younger patients are more prone to use efficiently new technologies.

In conclusion, results of this study indicate that sc IFN β-1a administration facilitated by RebiSmart^®^ in patients that accepted it, could lead to high rates of adherence to a prescribed dose regimen over 36 months. This adherence was above 90% on average over the three years of use. Advances in electronic injection devices such as RebiSmart^®^ permit adherence to be recorded and monitored by the device itself, thereby improving comfort, subject satisfaction, and the adherence to the treatment of patients in this chronic disease.

## References

[1] Compston A, Coles A. Multiple sclerosis. Lancet. 2008; 372: 1502–1517. 10.1016/S0140-6736(08)61620-7 18970977

[2] Noseworthy JH, Lucchinetti C, Rodriguez M, Weinshenker BG. Multiple sclerosis. N Engl J Med. 2000; 343: 938–995. 11006371 10.1056/NEJM200009283431307

[3] Fernández O, Fernández V, Guerrero M, León A, López-Madrona JC, Alonso A, et al Multiple sclerosis prevalence in Malaga, Southern Spain estimated by the capture-recapture method. Mult Scler. 2012;18:372–376. 10.1177/1352458511421917 21878452

[4] Bártulos Iglesias M, Marzo Sola ME, Estrella Ruiz LA, Bravo Anguiano Y. Epidemiological study of multiple sclerosis in La Rioja. Neurologia. 2014: S0213-4853(14)00124-8. 10.1016/j.nrl.2014.04.016 24975346

[5] Weinshenker BG, Bass B, Rice GP, Noseworthy J, Carriere W, Baskerville J, et al The natural history of multiple sclerosis: a geographically based study. I. Clinical course and disability. Brain. 1989; 112: 133–146. 2917275 10.1093/brain/112.1.133

[6] IFNB Multiple Sclerosis Study Group. Interferon beta-1b is effective in relapsing-remitting multiple sclerosis. I. Clinical results of a multicenter, randomized, double-blind, placebo-controlled trial. The IFNB Multiple Sclerosis Study Group. Neurology. 1993; 43: 655–661. 8469318 10.1212/wnl.43.4.655

[7] Jacobs LD, Cookfair DL, Rudick RA, Herndon RM, Richert JR, Salazar AM, et al Intramuscular interferon beta-1a for disease progression in relapsing multiple sclerosis. The Multiple Sclerosis Collaborative Research Group (MSCRG). Ann Neurol. 1996; 39: 285–294. 8602746 10.1002/ana.410390304

[8] Johnson KP, Brooks BR, Cohen JA, Ford CC, Goldstein J, Lisak RP, et al Copolymer 1 reduces relapse rate and improves disability in relapsing-remitting multiple sclerosis: results of a phase III multicenter, double-blind placebo-controlled trial. The Copolymer 1 Multiple Sclerosis Study Group. Neurology. 1995; 45: 1268–1276. 7617181 10.1212/wnl.45.7.1268

[9] PRISMS Study Group. Randomised double-blind placebo-controlled study of interferon beta-1a in relapsing/remitting multiple sclerosis. PRISMS (Prevention of Relapses and Disability by Interferon beta-1a Subcutaneously in Multiple Sclerosis) Study Group. Lancet. 1998; 352: 1498–1504. 9820297

[10] Tremlett HL, Oger J. Interrupted therapy: stopping and switching of the beta-interferons prescribed for MS. Neurology. 2003; 61: 551–554. 12939437 10.1212/01.wnl.0000078885.05053.7d

[11] Treadaway K, Cutter G, Salter A, Lynch S, Simsarian J, Corboy J, et al Factors that influence adherence with disease-modifying therapy in MS. J Neurol. 2009; 256: 568–576. 10.1007/s00415-009-0096-y 19444532

[12] Costello K, Kennedy P, Scanzillo J. Recognizing nonadherence in patients with multiple sclerosis and maintaining treatment adherence in the long term. Medscape J Med. 2008; 10: 225 19008986 PMC2580090

[13] Al-Sabbagh A, Bennet R, Kozma C, Dickson M, Meletiche D. Medication gaps in disease-modifying therapy for multiple sclerosis are associated with an increased risk of relapse: findings from a national managed care database. J Neurol. 2008; 255: S79.

[14] Steinberg SC, Faris RJ, Chang CF, Chan A, Tankersley MA. Impact of adherence to interferons in the treatment of multiple sclerosis: a nonexperimental, retrospective, cohort study. Clin Drug Investig. 2010; 30: 89–100. 10.2165/11533330-000000000-00000 20067327

[15] Devonshire V, Lapierre Y, Macdonell R, Ramo-Tello C, Patti F, Fontoura P, et al The Global Adherence Project (GAP): a multicenter observational study on adherence to disease-modifying therapies in patients with relapsing-remitting multiple sclerosis. Eur J Neurol. 2011; 18: 69–77. 10.1111/j.1468-1331.2010.03110.x 20561039

[16] Fernández O, Agüera E, Izquierdo G, Millán-Pascual J, Ramió I Torrentà L, Oliva P, et al Adherence to interferon β-1b treatment in patients with multiple sclerosis in Spain. PLoS One. 2012; 7: e35600 10.1371/journal.pone.0035600 22615737 PMC3353967

[17] Mohr DC, Boudewyn AC, Likosky W, Levine E, Goodkin DE. Injectable medication for the treatment of multiple sclerosis: the influence of self-efficacy expectations and injection anxiety on adherence and ability to self-inject. Ann Behav Med. 2001; 23: 125–132. 11394554 10.1207/S15324796ABM2302_7

[18] Cox D, Stone J. Managing self-injection difficulties in patients with relapsing-remitting multiple sclerosis. J Neurosci Nurs. 2006; 38: 167–171. 16817668 10.1097/01376517-200606000-00005

[19] Beer K, Muller M, Hew-Winzeler AM, Bont A, Maire P, You X, et al The prevalence of injection-site reactions with disease-modifying therapies and their effect on adherence in patients with multiple sclerosis: an observational study. BMC Neurol. 2011; 11: 144 10.1186/1471-2377-11-144 22074056 PMC3227610

[20] Exell S, Verdun E, Driebergen R. A new electronic device for subcutaneous injection of IFN beta-1a. Expert Rev Med Devices. 2011; 85: 543–553. 10.1586/erd.11.29 21728909

[21] Lugaresi A. RebiSmart™ (version 1.5) device for multiple sclerosis treatment delivery and adherence. Expert Opin Drug Deliv. 2013; 10: 273–283. 10.1517/17425247.2013.746311 23252744

[22] Lugaresi A, Florio C, Brescia-Morra V, Cottone S, Bellantonio P, Clerico M, et al Patient adherence to and tolerability of self-administered interferon β-1a using an electronic autoinjection device: a multicentre, open-label, phase IV study. BMC Neurol. 2012; 12: 7 10.1186/1471-2377-12-7 22390218 PMC3368780

[23] Willis H, Webster J, Larkin AM, Parkes L. An observational, retrospective, UK and Ireland audit of patient adherence to subcutaneous interferon beta-1a injections using the RebiSmart(®) injection device. Patient Prefer Adherence. 2014; 8: 843–851. 10.2147/PPA.S54986 24966669 PMC4062562

[24] Brown MG, Kirby S, Skedgel C, Fisk JD, Murray TJ, Bhan V, et al How effective are disease-modifying drugs in delaying progression in relapsing-onset MS? Neurology. 2007; 69: 1498–1507. 17699802 10.1212/01.wnl.0000271884.11129.f3

[25] Bayas A. Improving adherence to injectable disease-modifying drugs in multiple sclerosis. Expert Opin Drug Deliv. 2013; 10: 285–287. 10.1517/17425247.2013.763793 23339371

[26] Seddighzadeh A, Hung S, Selmaj K, Cui Y, Liu S, Sperling B, et al Single-use autoinjector for peginterferon-β1a treatment of relapsing-remitting multiple sclerosis: safety, tolerability and patient evaluation data from the Phase IIIb ATTAIN study. Expert Opin Drug Deliv. 2014; 11: 1713–1720. 10.1517/17425247.2014.944159 25073663

[27] Brochet B, Lemaire G, Beddiaf A. Reduction of injection site reactions in multiple sclerosis (MS) patients newly started on interferon beta 1b therapy with two different devices. Rev Neurol (Paris). 2006; 162: 735–740. 16840982 10.1016/s0035-3787(06)75071-8

[28] Cramer JA, Cuffel BJ, Divan V, Al-Sabbagh A, Glassman M. Patient satisfaction with an injection device for multiple sclerosis treatment. Acta Neurol Scand. 2006; 113: 156–162. 16441244 10.1111/j.1600-0404.2005.00568.x

[29] Mikol D, Lopez-Bresnahan M, Taraskiewicz S, Chang P, Rangnow J; Rebiject Study Group. A randomized, multicentre, open-label, parallel-group trial of the tolerability of interferon beta-1a (Rebif^®^) administered by autoinjection or manual injection in relapsing-remitting multiple sclerosis. Mult Scler. 2005; 11: 585–591. 16193898 10.1191/1352458505ms1197oa

[30] de Sá J, Urbano G, Reis L. Assessment of new application system in Portuguese patients with relapsing-remitting multiple sclerosis. Curr Med Res Opin. 2010; 26: 2237–2242. 10.1185/03007995.2010.508688 20687777

